# Supplementary material for: Analytical Performance of a Novel Latex Turbidimetric Immunoassay, “Nanopia TARC”, for TARC/CCL17 Measurement: A Retrospective Observational Study
Source: Diagnostics (Basel). 2023 Sep 13;13(18):2935. doi: 10.3390/diagnostics13182935 (PMC10529481; doi:10.3390/diagnostics13182935)
Supplement: Supplementary file 1 [file diagnostics-13-02935-s001.zip › diagnostics-2531129-supplementary.pdf]

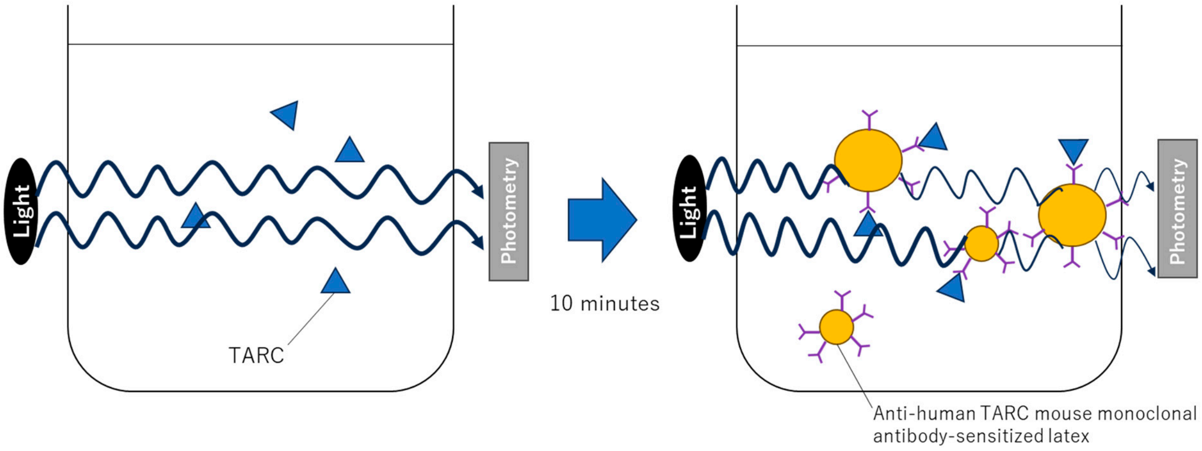

Figure S1. A novel latex turbidimetric immunoassay, "Nanopia TARC."

In this homogeneous assay, anti-human TARC mouse monoclonal antibody-sensitized latex is reacted with thymus and activation-regulated chemokine (TARC), and the absorbance is measured directly without separating the bound/free (B/F) molecules. It can be loaded onto a general clinical chemistry analyzer and provides results in 10 min.

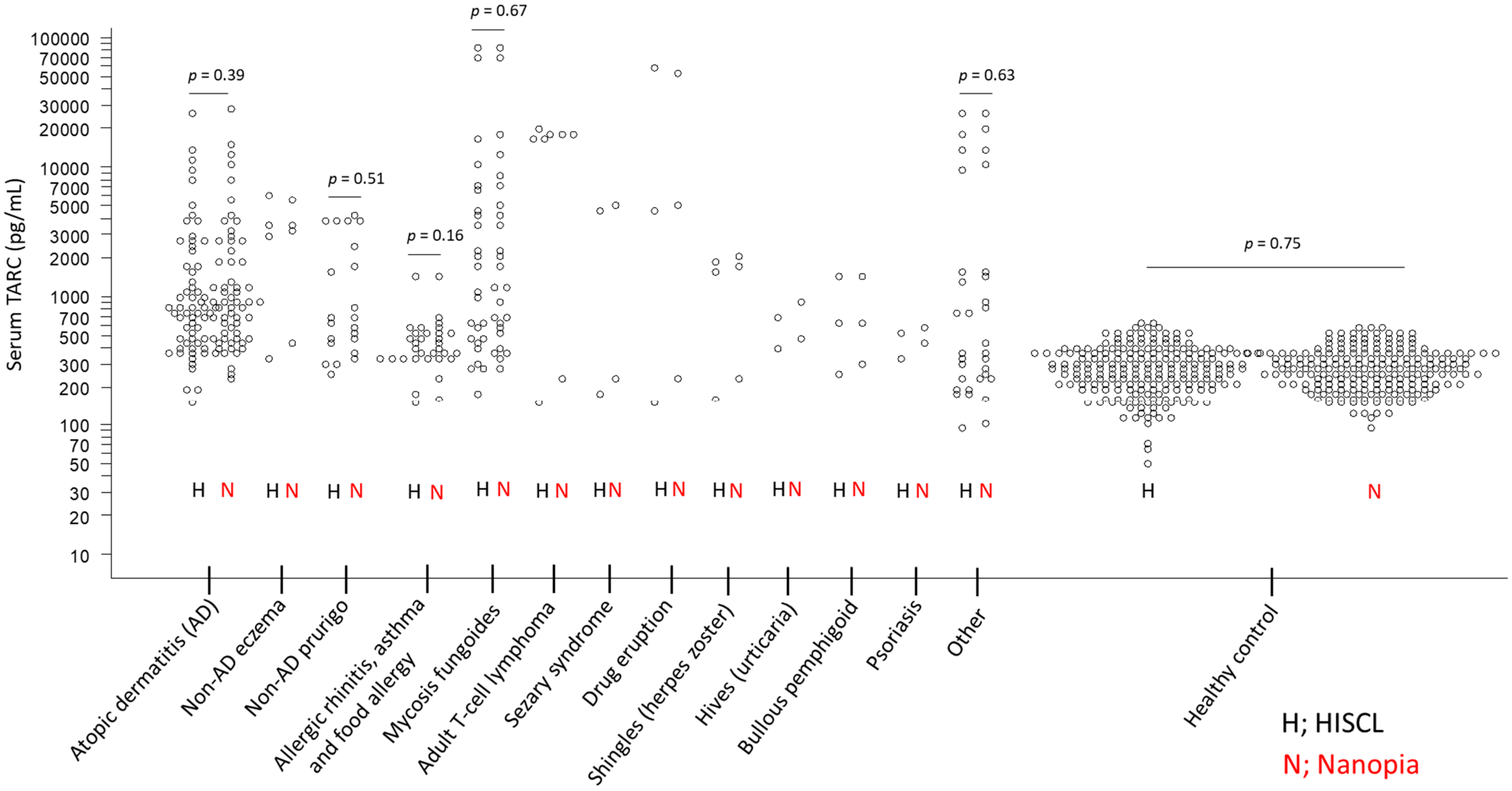

Figure S2. Distribution of the thymus and activation-regulated chemokine (TARC) levels measured for allergic and skin diseases. Dot plots show the distribution of TARC values determined using the two methods (N in red: Nanopia TARC; H in black: HISCL TARC). The Y-axis shows logarithms of serum TARC levels and the X-axis shows the different diseases. The p value represents the difference between the two methods in diseases with 10 or more cases (Mann–Whitney U test; significance level set at $p < 0.001$).

Table S1. Measurement conditions prescribed in the manufacturer's protocol for LABOSPECT008α.

Principle	Absorption spectroscopy
Method	2-point end assay
Reaction time	10 min
1st photometry	5.8 min
2nd photometry	10 min
Photometric wavelength (main-/sub-)	570/800 nm
Sample volume	2.0 μL
1st reagent volume	100 μL
2nd reagent volume	33 μL
Washing reagent	HICARRYNON (Citric acid monohydrate <10%, oxyethylene = alkyl ether 5%)
Calibration curve	Multi-point spline curve
